# Electrical Stimulation of the M1 Activates Somatostatin Interneurons in the S1: Potential Mechanisms Underlying Pain Suppression

**DOI:** 10.1523/ENEURO.0541-24.2025

**Published:** 2025-04-25

**Authors:** Junhee Park, Yong Geon Kim, Taehyeon Kim, Myungin Baek

**Affiliations:** Department of Brain Sciences, DGIST, Daegu 42988, Republic of Korea

**Keywords:** analgesic effect, electrical stimulation, neuropathic pain, primary motor cortex, primary somatosensory cortex, somatostatin interneurons

## Abstract

Chronic pain affects millions globally, yet no universally effective treatment exists. The primary motor cortex (M1) has been a key target for chronic pain therapies, with electrical stimulation of the M1 (eMCS) showing promise. However, the mechanisms underlying M1-mediated analgesic effects are not fully understood. We investigated the role of the primary somatosensory cortex (S1) in M1-mediated analgesia using a neuropathic pain mouse model. In this model, neuropathic pain is associated with increased spontaneous activity of layer V pyramidal neurons (LV-PNs) in the S1, partly attributed to the reduced activity of somatostatin-expressing inhibitory neurons (SST^+^ INs), which normally suppress LV-PNs. While manipulation of either LV-PNs or SST^+^ INs has been shown to alleviate pain, the role of S1 in M1-mediated analgesia has not been identified. Using multichannel silicon probes, we applied eMCS to neuropathic mice and observed significant analgesia. Histological analyses revealed that eMCS activated SST^+^ INs and suppressed hyperactivity of LV-PNs in the S1, suggesting that eMCS suppresses pain by modulating S1 neuronal circuits, alongside other pain-related regions. Notably, eMCS induced long-lasting analgesia, persisting for at least 2 d poststimulation. These findings implicate S1 as a critical mediator of eMCS-induced analgesia and suggest eMCS as a potential durable therapeutic strategy for chronic pain.

## Significance Statement

Chronic pain is a devastating disorder that affects over 25% of the global population. The lack of universally and entirely effective treatments, combined with severe social and economic burdens posed by the side effects of current analgesics, underscores the need to explore multifaceted approaches. In this study, we applied a silicon probe to target layer 5 of the M1 region of mice and delivered electrical stimulation to a chronic constriction injury mouse model. Our findings demonstrated that eMCS induced analgesic effects on mechanical stimuli, with the effect notably persisting for at least 2 d after the cessation of eMCS. As a potential mechanism, we identified SST+ neuronal activation in S1, along with other previously known brain regions activated by MCS.

## Introduction

Chronic pain is a debilitating disorder affecting a vast number of people globally with largely unidentified causes, presenting significant social challenges (Costigan et al., 2009; Colloca et al., 2017). As of now, there is not a universally effective treatment method for chronic pain. Since the analgesic effects of eMCS were reported (Tsubokawa et al., 1991a,b), diverse stimulation methods have been tested in the M1 of both model organisms and humans (Pleger et al., 2004; Bachmann et al., 2010; Ramos-Fresnedo et al., 2022). However, the underlying mechanisms remain elusive. Using chemo- and optogenetic stimulation of the M1 (chopMCS), the direct targets of the M1 that mediate analgesic effect were identified. Neurons in the zona incerta (ZI) and periaqueductal gray (PAG) areas, which are well known for their involvement in controlling pain responses (Hosobuchi et al., 1977; Behbehani, 1995; Masri et al., 2009; Bachmann et al., 2010; Kim et al., 2018; Lu et al., 2021; Singh et al., 2022), were activated by chopMCS on LV-PNs in the hindlimb area of the M1 (Gan et al., 2022). Similarly, opto- and chemogenetic stimulation of excitatory neurons in the ZI and PAG that are directly connected with LV-PNs in the hindlimb area of the M1 dampens the pain response.

Beyond the ZI and PAG, LV-PNs in the M1 form connections throughout the central nervous system (Munoz-Castaneda et al., 2021; Zhang et al., 2021), including the anterior cingulated cortex (ACC) and the S1. Both the ACC and S1 are critically implicated in pain regulation. The ACC and S1 can control pain responses through direct descending inputs to pain circuits in the spinal cord (Bushnell et al., 2013; Liu et al., 2018; Chen and Heinricher, 2019; Dougherty and Ha, 2019). Inhibition or activation of neurons in the ACC that project directly to the spinal cord have been found to reduce and increase pain responses, respectively (Chen et al., 2018). Similarly, silencing PNs in the S1 or transecting the pyramidal tract has been shown to reduce pain responses (Atasoy et al., 2012; Liu et al., 2018).

However, the ACC and S1 exhibited no changes in the number of activated neurons in response to chopMCS (Gan et al., 2022). Despite this, the fact that some neurons in the ACC and S1 were still active during M1 stimulation (Gan et al., 2022), that there is a significant heterogeneity of neuronal subtypes in the cortex including excitatory and inhibitory neurons (Naskar et al., 2021), and that manipulation of different sets of neurons in the same brain region resulted in varied pain responses (Gu et al., 2015; Chen et al., 2018) led us to hypothesize that there may be differences in the composition, but not in the number, of activated neurons in the S1 following M1 stimulation compared with control such that M1 stimulation might induce its analgesic effects by selectively modulating specific neuronal subpopulations within the S1, rather than altering overall activity levels.

Further supporting the need to investigate specific neuronal subtypes, differential neuronal responses have been reported in the S1 of the mouse pain models, necessitating specific regulation of neuronal subtypes for effective pain control. Specifically, LV-PNs exhibit increased spontaneous activities in chronic pain mouse models (Cichon et al., 2017; Okada et al., 2021). The heightened activity of LV-PNs has a causal relationship with pain response. Inhibition of PNs or activation of SST^+^ INs, which inhibit LV-PNs activity, has been shown to reduce chronic pain responses (Cichon et al., 2017; Okada et al., 2021). This increase in the PNs activity is partly attributed to reduced activity of SST^+^ INs, which is mediated by stronger inhibition from vasoactive intestinal peptide-positive (VIP^+^) INs (Ding et al., 2023).

To investigate S1 involvement in M1 stimulation-induced analgesia, we applied an electrical stimulation setup with potential translational applications. Using a silicon probe to stimulate a specific M1 layer, we stimulated layer V and induced an analgesic effect in a chronic constriction injury mouse model through a long duration eMCS under mild stimulation conditions. Histological experiments revealed that SST^+^ INs among inhibitory interneurons were especially activated in the S1 following eMCS, with a concomitant decrease in the activity of LV-PNs. This finding suggests that eMCS-induced activation of SST^+^ INs in the S1 suppress the increased spontaneous activities in S1 LV-PNs, a contributing factor to neuropathic pain responses. Not reported in chopMCS, eMCS induced a prolonged analgesic effect even after cessation of stimulation, suggesting plasticity changes in the S1. These findings highlight the unique ability of eMCS to modulate the S1 and induce long-lasting pain relief.

This study provides further insight into the analgesic effects of eMCS, potentially opening new avenues for chronic pain management in humans. By revealing activity changes in specific S1 neuronal populations, we contribute to a more comprehensive understanding of pain modulation mechanisms and the therapeutic potential of M1 stimulation.

## Materials and Methods

### Animals

All methods employed in this manuscript are reported in accordance with ARRIVE guidelines (https://arriveguidelines.org). All experiments were approved by the Institutional Animal Care and Use Committee (IACUC) of DGIST (Approval No: DGIST-IACUC-21090806-0000). All procedures were conducted in accordance with approved protocols and laboratory safety guidelines of DGIST. Every effort was made to minimize the number of animals used and their discomfort in all experiments. Adult male mice (C57BL/6J, 8 weeks old) were sourced from a local breeding facility (Hyochang Science). Mice were maintained under a 12 h light/dark cycle at a controlled temperature (22 ± 1°C) with 40–60% humidity. They had access to food and water *ad libitum*. Anesthesia was induced using 2.5% isoflurane in oxygen and following surgical procedures were conducted on a stereotaxic frame (RWD). The eyes were protected with eye ointment (Liposic) during surgery. The surgical site was sutured using either autoclip or sterile silk sutures (7-0 Black Silk). Dental cement-based crowns, Super Bond C&B (Sun Medical), and Jet Denture Repair (Lang) were utilized to secure the head post and grounding.

### Neuropathic pain induction

Neuropathic pain was induced using the Chronic Constriction Injury (CCI) model on the sciatic nerve. CCI was induced following the method previously described (Bennett and Xie, 1988). Adult male mice were deeply anesthetized with isoflurane in oxygen (2.5 volume%), and the right common sciatic nerve was exposed at the mid-thigh level. Three ligatures (7-0 Black Silk) were loosely tied around the right common sciatic nerve at 1 mm intervals, such that they were only tightened to a point where a slight tremor was observed in the right hindlimb. The surgical incision was closed in two layers. Sham-treated mice underwent a similar surgical procedure but without nerve ligation, where the skin was incised and subsequently sutured. After surgery, mice were allowed to recover under a heat lamp.

### Electrode implantation

Adult male mice were anesthetized with 2.5 volume% isoflurane in oxygen. Under a stereotaxic apparatus, the skull was exposed to position a commercial multichannel silicon probe (A4 × 4-3mm-100-125-703-CM16LP, NeuroNexus) on the hindlimb motor cortex (1.0 mm rostral and 1.0 mm lateral to the bregma at a depth of 0.9 mm from the cortical surface). To avoid electrode interference, two holes with a diameter of 1 mm, located 3–4 mm apart, were drilled. Two stainless steel screws (1 mm diameter and 4 mm length) were subsequently inserted to serve as ground and reference, as well as to secure the setup. The exposed skull and the implanted electrode were then covered with n-butyl cyanoacrylate adhesive and dental cement for securement.

### Electrical stimulation of the M1

Under brief anesthesia with 2.5 volume% isoflurane in oxygen, the electrode was connected to an electrode headstage (Intan, RHS 16-channel stim/recording headstage). After awakening from the anesthesia, which took ∼10 min, electrical stimulation was administered to the freely moving animal for 30 min using a stimulator (Intan, RHS's 128-channel stimulation/recording controller). The stimulation setup was designed using Intan RHX data acquisition software, with the following parameters: pulse train, 20 ms at 50 Hz; poststimulation refractory period, 1 ms; stimulation waveform, biphasic with delay and cathodic current first (first phase duration: 200 μs; interphase delay, 200 μs; second phase duration, 200 μs; amplitudes, 5 μA). The intensity of the electrical stimulation applied was adjusted to ensure that it did not interfere with the animals’ general behaviors during the procedure.

### Von Frey assay

Adult male mice were acclimated on a wire-mesh platform in an acrylic chamber 1 h before the test. Mechanical hypersensitivity was assessed by applying force to the plantar surface of the paw. The bending or licking behaviors were considered as a response to the stimulation. A 0.4 g filament (Stoelting, 58011) was initially used to deliver the force. The “up-down” method (Chaplan et al., 1994) was employed for the von Frey assay. In the absence of a response to a given stimulus, the next higher microfilament was applied; conversely, if there was a response, the next lower microfilament was introduced. The experiments were conducted under randomized and blinded conditions.

### RNA in situ hybridization

Tissues from the same mice used to measure withdrawal thresholds were collected for histological analysis. Tissues were prepared as follows: mice were perfused with ice-cold 4% paraformaldehyde (PFA) under anesthesia [2% solution of 2,2,2-tribromoethanol (Sigma-Aldrich, T48402-25G) in 2-methyl-2-butanol (Sigma-Aldrich, 240486-100ML)]. Tissues were postfixed in 4% PFA overnight at 4°C, followed by four washes in cold PBS for 15 min each, and then incubated overnight in 30% sucrose. Tissues embedded in OCT were frozen on dry ice and sectioned at a thickness of 30 μm using a cryostat (Leica, CM3050S). RNA in situ hybridization was performed as previously described (Jung et al., 2018): in brief, tissue sections were dried for 1 h at RT, fixed in 4% PFA for 10 min at RT. Slides were treated with Proteinase K solution (1 μg/ml) for 5 min at RT. Slides were acetylated to block positive charges in tissue. A total of 100 μl of hybridization solution containing 100 ng of DIG-labeled antisense *c-fos* probes (*c-fos* probe 1 and *c-fos* probe 2, 50 ng each) was applied to each slide. Antibody solution containing 1% heat inactivated goat serum and a 1:5,000 dilution of anti-DIG-AP antibody (Roche-Aldrich, catalog #11093274910) was applied to the slide and incubated overnight at 4°C. Slides were then incubated overnight at 4°C in a humidified chamber. The following day, slides were washed three times 5 min each with 0.75 ml/slide of buffer B1 (0.1 M Tris, pH 7.5, 150 mM NaCl). Slides were then transferred to buffer B3 (0.1 M Tris, pH 9.5, 100 mM NaCl, 50 mM MgCl_2_) and incubated for 5 min. Signals were detected by applying a solution containing 3.5 μl/ml BCIP (Roche, catalog #1383221001) and 3.5 μl/ml NBT (Roche, catalog #1383213001) in buffer B3. DIG-labeled *c-fos* probes were generated using the following primers (Carvalho et al., 2015; 5′ -> 3′):

*c-fos* probe1 forward primer: CAG CGA GCA ACT GAG AAG AC; *c-fos* probe1 reverse primer: TAA TAC GAC TCA CTA TAG GGG CTG CAT AGA AGG AAC CGG AC; *c-fos* probe2 forward primer: GGA GCC AGT CAA GAG CAT CAG; *c-fos* probe2 reverse primer: TAA TAC GAC TCA CTA TAG GGA ATG AAC ATT GAC GCT GAA GGA C

### Multiplex RNAscope in situ hybridization

Fluorescent in situ hybridization was performed to detect different RNA expression simultaneously. Multiplex RNA in situ hybridization was performed following the manufacturer's instruction (RNAscope Multiplex Fluorescent Reagent Kit v2; ACDBio, catalog #323100) with some modifications: slides were treated with xylene for 8 min at RT and antigen retrieval was performed in 1× target retrieval reagent for 5 min at 90°C. The following probes were used to detect gene expression: Mm-Fos-C1 (ACDBio, catalog #316921-C1); Mm-Slc17a6-C2 (ACDBio, catalog #319171-C2); Mm-Slc17a7-O2-C2 (ACDBio, catalog #501101-C2); Mm-Sst-C2 (ACDBio, catalog #404631-C2); Mm-Gad1-C3 (ACDBio, catalog #400951-C3). Fluorophores used to detect probes were as follows: C1 probes, Opal 570 (Akoya Biosciences, catalog #FP1488001KT); C2 probes, Opal 520 (Akoya Biosciences, catalog #FP1487001KT); C3 probes, Opal 690 (Akoya Biosciences, catalog #FP1497001KT).

### Image acquisition

Multiplex RNAscope in situ hybridization images were acquired using a confocal microscope (Zeiss, LSM800) with a 10× objective. Chromogenic in situ hybridization images were acquired using a light microscope (Leica, DM500). All acquired images were subsequently processed using Fiji (Schneider et al., 2012).

### Quantification of *c-Fos* expression

*c-fos* expression was quantified manually under blinded conditions. All slides were stained under identical experimental conditions and imaged using consistent confocal settings for the same probe sets. The images were then processed in Fiji. *c-fos* expression was counted when the signal overlapped by >50% with DAPI, *Vglut1*, *Gad1*, *Sst*, or *Vglut2* signals. Brain regions and cortical layers, as well as Rexed laminae in the dorsal horn of the spinal cord, were defined according to established reference atlases (Allen Institute for Brain Science, 2011; Paxinos et al., 2019).

### Behavioral analysis

For behavioral experiments during eMCS, mice were habituated in their individual housing cages for at least 30 min before the procedure. Videos were recorded using a monochrome camera (The Imaging Source, DMK 33UX273) and processed with IC Capture. Video recordings were performed for ∼5 min prior to eMCS and continued for ∼30 min during eMCS. Animal movements were tracked using idtracker.ai (5.2.12; Romero-Ferrero et al., 2019). Movement parameters, including distance traveled per min, average speed, and moving/resting ratios, were calculated. Resting was defined as periods when the mouse moved <1 mm, while moving was defined as periods with movement exceeding 1 mm. The proportion of resting was determined by analyzing the proportion of frames classified as moving or resting within the total frames. For more detailed analysis, trajectories were normalized to the cage dimensions (165 mm × 403 mm) and visualized with *x*- and *y-*coordinates adjusted to the fixed scale. All analyses were performed using Python (3. 12. 9).

### Graphics and statistical analysis

All figures were composed and adjusted using Adobe Illustrator (Adobe). All statistical analyses were performed using GraphPad Prism (ver. 8.4.3).

## Results

### eMCS reduces neuropathic pain response

**Figure 1.**
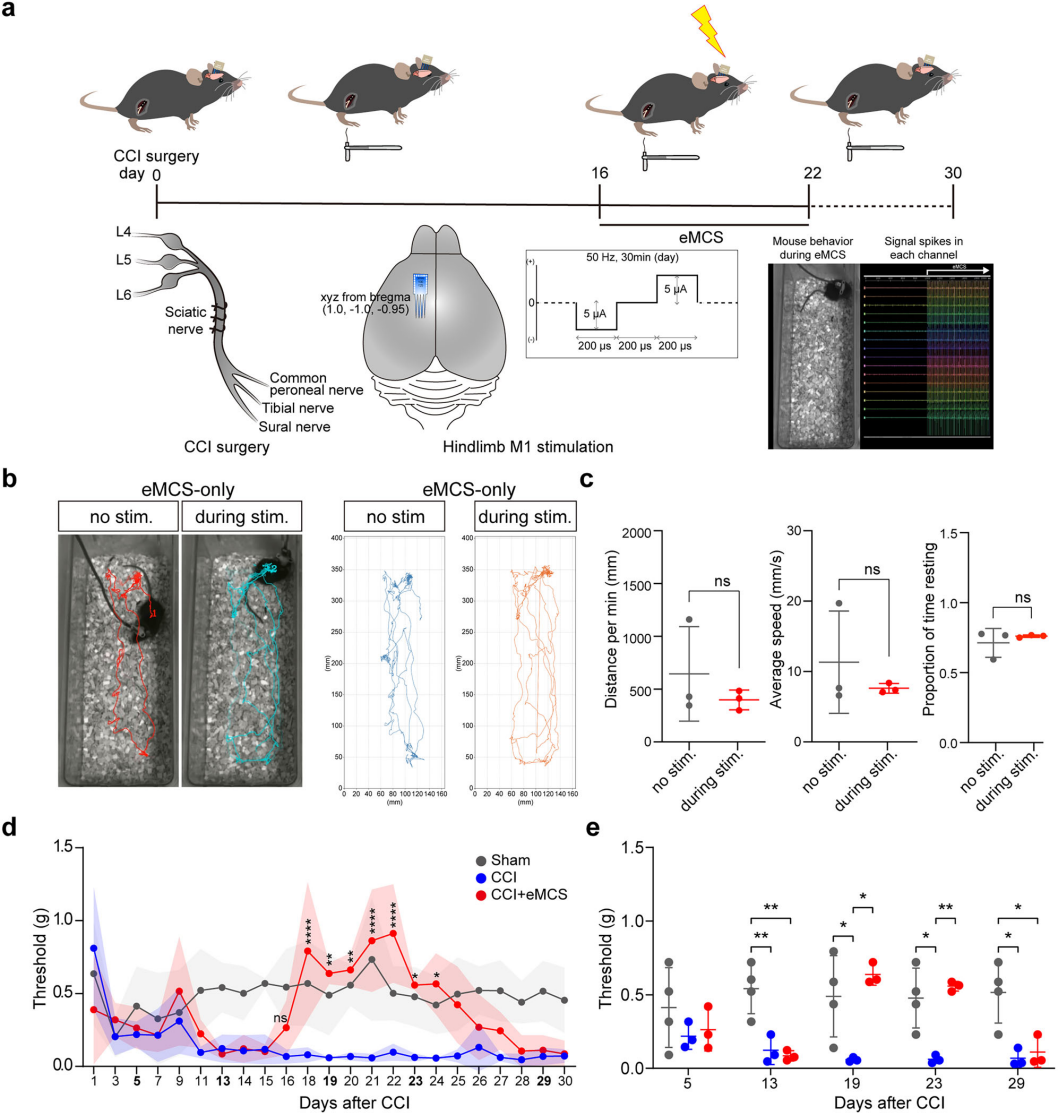
eMCS suppresses pain responses. ***a***, Schematic summary of the experiment. Bottom right, Animal behavior video capture (left) during eMCS (right). ***b***, Overall behavioral effects of eMCS. Representative trajectory plots showing mouse movement in a cage with or without stimulation. eMCS was provided 1–2 weeks after electrode implantation in the M1. Animal behavior was video-taped for ∼5 min (without stimulation, eMCS-only) and 30 min (with stimulation, eMCS-only). An equal number of video frames, corresponding 2–5 min interval, were used for comparison between groups. ***c***, Quantification of general locomotion. Sham (*n* = 3); eMCS-only (*n* = 3). Left, Distance traveled per minute (mm/min). Middle, Average speed (mm/s). Right, Proportion of time spent resting. Lines indicate the mean ± SD. ns, not significant; paired *t* test. ***d***, Mechanical sensitivity determined by force required for 50% threshold for paw withdrawal, as determined through von Frey assay. Sham, skin incision only; CCI, CCI surgery; CCI + eMCS, eMCS with CCI surgery. Sham group (*n* = 4); CCI group (*n* = 3); CCI + eMCS group (*n* = 3). Lines are the mean ± standard deviation (SD). eMCS was performed from Day 16 to Day 22. Statistical test between CCI group and CCI + eMCS group: ns, not significant; *p < 0.05; **p < 0.01; ***p < 0.001; ****p < 0.0001; two-way ANOVA followed by Tukey's test. ***e***, Quantification of the pain response based on withdrawal thresholds to mechanical stimuli in ***b*** across days postsurgery. Day 5 and Day 13, pre-eMCS; Day 19, eMCS; Day 23 and Day 29, post-eMCS. Sham group (*n* = 4); CCI group (*n* = 3); CCI + eMCS group (*n* = 3). Lines indicate the mean ± SD. Only significant differences are indicated: *p < 0.05; **p < 0.01; one-way ANOVA followed by Tukey's test. See also Extended Data Figure 1-1.

We inserted a multichannel silicon probe into the hindlimb area of the M1 (Li and Waters, 1991; Pronichev and Lenkov, 1998; Fig. 1*a*) to stimulate neurons in layer V, from which the analgesic effects originate (Gan et al., 2022). Simultaneously, we performed surgery on the sciatic nerve of the hindlimb to create a chronic constriction injury (CCI) model (Bennett and Xie, 1988; van der Wal et al., 2015), a well-known neuropathic pain model. One group of mice received only CCI surgery and served as the CCI group, while another group received both CCI and probe implantation, constituting the CCI + eMCS group. As for the sham group, a separate set of mice underwent surgery involving an incision on the skin of the hindlimb.

10.1523/ENEURO.0541-24.2025.f1-1 Figure 1-1 **Two-way ANOVA test of von Frey assay.** Mechanical sensitivity determined by force required for 50% threshold for paw withdrawal, as determined through von Frey assay. eMCS was performed from Day 16 to Day 22. Sham: skin incision only; CCI: CCI surgery; CCI + eMCS: eMCS with CCI surgery. Sham group (n = 4); CCI group (n = 3); CCI + eMCS group (n = 3). A statistical test was performed between groups on each day post-surgery: ns, not significant; *p < 0.05; **p < 0.01; ***p < 0.001; ****p < 0.0001; two-way ANOVA followed by Tukey's test. Download Figure 1-1, DOC file.

The electrical strength and duration were adjusted to avoid obvious behavioral responses during stimulation. The general locomotion was not changed before and during eMCS (Fig. 1*b*,*c*). Mechanical pain response was measured using von Frey assay (Chaplan et al., 1994). To confirm successful induction of the neuropathic pain model, measurements were taken starting on postsurgery day 1 (ps1). We identified the time at which the mechanical response reached maximum sensitivity, approximately ps13 after CCI surgery in both the CCI and CCI + eMCS groups (Fig. 1*d*, Extended Data Fig.1-1; ps13 threshold mean ± SD: sham, 0.54 ± 0.17; CCI, 0.12 ± 0.10; CCI + eMCS, 0.08 ± 0.03). To ensure that sensitivity had peaked, we waited until the pain response remained stable for 3 consecutive days. Once the pain response was consistently elevated for 3 consecutive days (Fig. 1*d*, Extended Data Fig. 1-1; ps13–15), electrical stimulation (amplitude, bipolar, 5 µA; duration, 200 µs; interval, 200 µs; frequency, 50 Hz) was administered daily to the CCI + eMCS group for 30 min until mechanical responses stabilized for an additional 3 consecutive days (Fig. 1*d*, Extended Data Fig. 1-1; ps20–22). While sensitivity remained high in the CCI group, the CCI + eMCS group recovered to levels comparable with those of the sham group (Fig. 1*d*,*e*; Extended Data Fig. 1-1; ps19 threshold mean ± SD: sham, 0.49 ± 0.28; CCI, 0.06 ± 0.02; CCI + eMCS, 0.64 ± 0.07). Finally, we ceased electrical stimulation and monitored the duration of the analgesic effect. For at least 2 d after discontinuing stimulation, sensitivity remained lower in the CCI + eMCS group compared with the CCI group (Fig. 1*d*); subsequently, sensitivity returned to levels similar to those of the CCI group.

### eMCS activates pain-related brain regions

**Figure 2.**
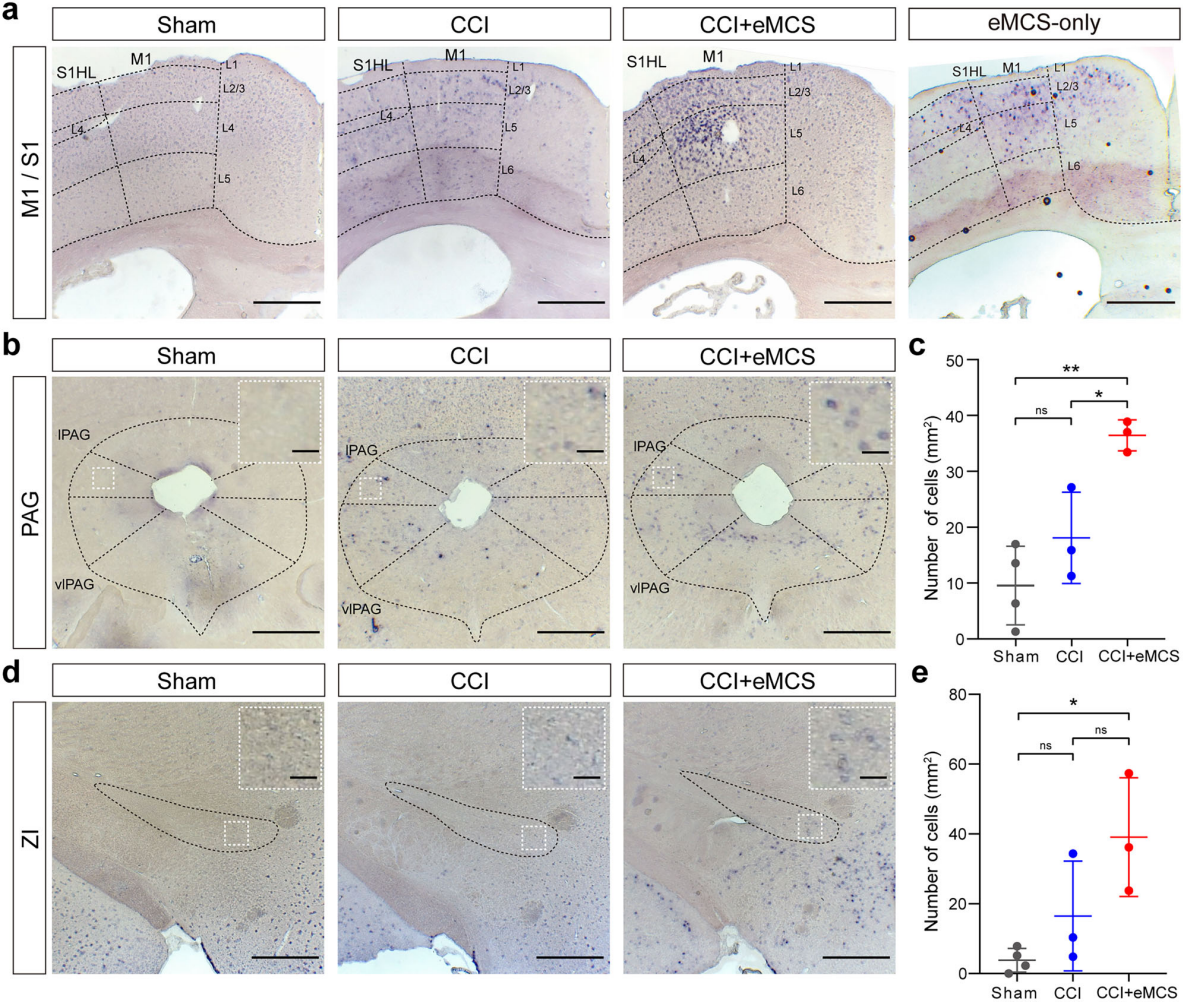
eMCS activates neurons in brain region related to pain suppression. ***a***, ***c***, ***d***, Representative images of *c-fos* in situ hybridization in the cortex (***a***), PAG (***c***), and ZI (***e***). ***a***, Shown from left to right: sham, CCI, CCI + eMCS, eMCS-only groups. Dashed lines, cortical region and layers. ***c***, ***d***, Shown from left to right: sham (left), CCI (middle), and CCI + eMCS (right), eMCS-only groups. Insets in the top right corner: magnified images of the regions marked with white dashed squares. Sham, skin incision only; CCI, CCI surgery; CCI + eMCS, eMCS with CCI surgery; eMCS-only, eMCS without CCI surgery. Scale bars: 500 µm (50 µm in insets). ***c***, ***e***, Quantification of the number of *c-fos*^+^ cells in the l/vlPAG (***c***) and ZI (***e***). Sham group (*n* = 4); CCI group (*n* = 3); CCI + eMCS group (*n* = 3). Lines indicate the mean ± SD. ns, not significant; *p < 0.05; **p < 0.01; one-way ANOVA followed by Tukey's test. lPAG, lateral periaqueductal gray; vlPAG, ventrolateral periaqueductal gray; ZI, zona incerta. See also Extended Data Figures 2-1, 2-2.

To identify regions mediating the analgesic effect of eMCS, we searched for the areas that were activated by eMCS. Neuronal activation was identified using the expression of *c-fos*, an early neuronal activation marker (Sagar et al., 1988). We performed RNA in situ hybridization experiments using probes against *c-fos* mRNA. In the CCI + eMCS group, we induced *c-fos* expression by applying the same electrical stimulation for 30 min without additional sensory stimuli before sacrificing the mice. Approximately 1 h after stimulation, the mice were perfused with 4% paraformaldehyde (PFA). The hindlimb area of the M1 contained a large number of cells expressing *c-fos* mRNA, mostly located in layer V (Fig. 2*a*), validating that electrical stimulation was properly targeted to the M1. In the spinal cord, we observed minimal differences or a slight increase in *c-fos* expression within the dorsal horn of the spinal cord among all three groups (Extended Data Fig. 2-1).

10.1523/ENEURO.0541-24.2025.f2-1 Figure 2-1 ***c-Fos* expression in the lumbar region of the spinal cord.** (**a**) Representative images of multiplex RNAscope *in situ* hybridization in the caudal lumbar region of the spinal cord (from left to right: sham, CCI, and CCI + eMCS). Red: *c-Fos*; green: *Vglut2*; blue: *Gad1*; grey: DAPI. Rexed laminae are outlined with white dashed lines. Sham: skin incision only; CCI: CCI surgery; CCI + eMCS: eMCS with CCI surgery. Scale bars: 200 µm. (**b**, **c**) Quantification of the number of *c-Fos*^+^ cells in the spinal cord. Total number of *c-Fos*^+^ cells (**b**) and the number of *c-Fos*^+^ cells in each Rexed lamina (**c**). Sham group (n = 2, 6 sections); CCI group (n = 2, 5 sections); CCI + eMCS group (n = 2, 6 sections). Lines indicate the mean ± SD. Only significant differences are indicated: *p < 0.05; one-way ANOVA followed by Tukey's test. Download Figure 2-1, TIF file.

10.1523/ENEURO.0541-24.2025.f2-2 Figure 2-2 ***c-Fos* expression in brain regions adjacent to the M1 region.** (**a**) Representative images of *c-Fos* expression in brain regions medial to the M1 region (M2 and ACC) across groups (from left to right: Sham, CCI, and CCI + eMCS). Red: *c-Fos*; blue: DAPI. Cortical layers are demarcated with white dashed lines. Sham: skin incision only; CCI: CCI surgery; CCI + eMCS: eMCS with CCI surgery. Scale bars: 500 µm. (**b**–**g**) Quantification of the number of *c-Fos*^+^ cells. Sham group (n = 4); CCI group (n = 3); CCI + eMCS group (n = 3). (**b**, **d,** and **f**) Total number of *c-Fos*^+^ cells in the M1 (**b**), M2 (**d**), and ACC (**f**). (**c**, **e**, and **g**) Number of *c-Fos*^+^ cells in each cortical layer in the M1 (**c**), M2 (**e**), and ACC (**g**). Lines indicate the mean ± SD. Only significant differences are indicated: *p < 0.05, **p < 0.01; one-way ANOVA followed by Tukey's test. Download Figure 2-2, TIF file.

Notably, while there were very few *c-fos*^+^ cells in the sham group in the cortex, *c-fos*^+^ cells were widely distributed in S1 of the CCI group (Fig. 2*a*), including layer V of S1, where pyramidal neurons are the predominant cell type, indicating increased spontaneous activity of pyramidal neurons as reported previously (Cichon et al., 2017; Okada et al., 2021; Wei et al., 2021). eMCS induced the number of *c-fos*^+^ cells in M1, with mostly enriched in layer 5 [Extended Data Fig. 2-2*a–c*; *c-fos*^+^ cells in layer 5 (mean ± SD): sham, 2.89 ± 2.30; CCI, 7.23 ± 9.12; CCI + eMCS, 40.07 ± 16.87].

Following eMCS, the PAG and ZI, which were previously shown to mediate the analgesic effects of chopMCS (Gan et al., 2022), exhibited an increased number of *c-fos+* cells compared with the sham group (Fig. 2*c–f*). The increase in *c-fos+* cells within the PAG was statistically significant compared with both the sham and CCI groups (mean ± SD: sham, 9.56 ± 7.06; CCI, 18.10 ± 8.17; eMCS, 36.45 ± 2.77). In contrast, the number of *c-fos+* cells in the ZI following eMCS showed a significant increase compared with the sham group, but not the CCI group (mean ± SD: sham, 3.82 ± 3.41; CCI, 16.52 ± 15.71; CCI + eMCS, 39.12 ± 16.99).

### eMCS increases the activity of inhibitory interneurons in the S1

**Figure 3.**
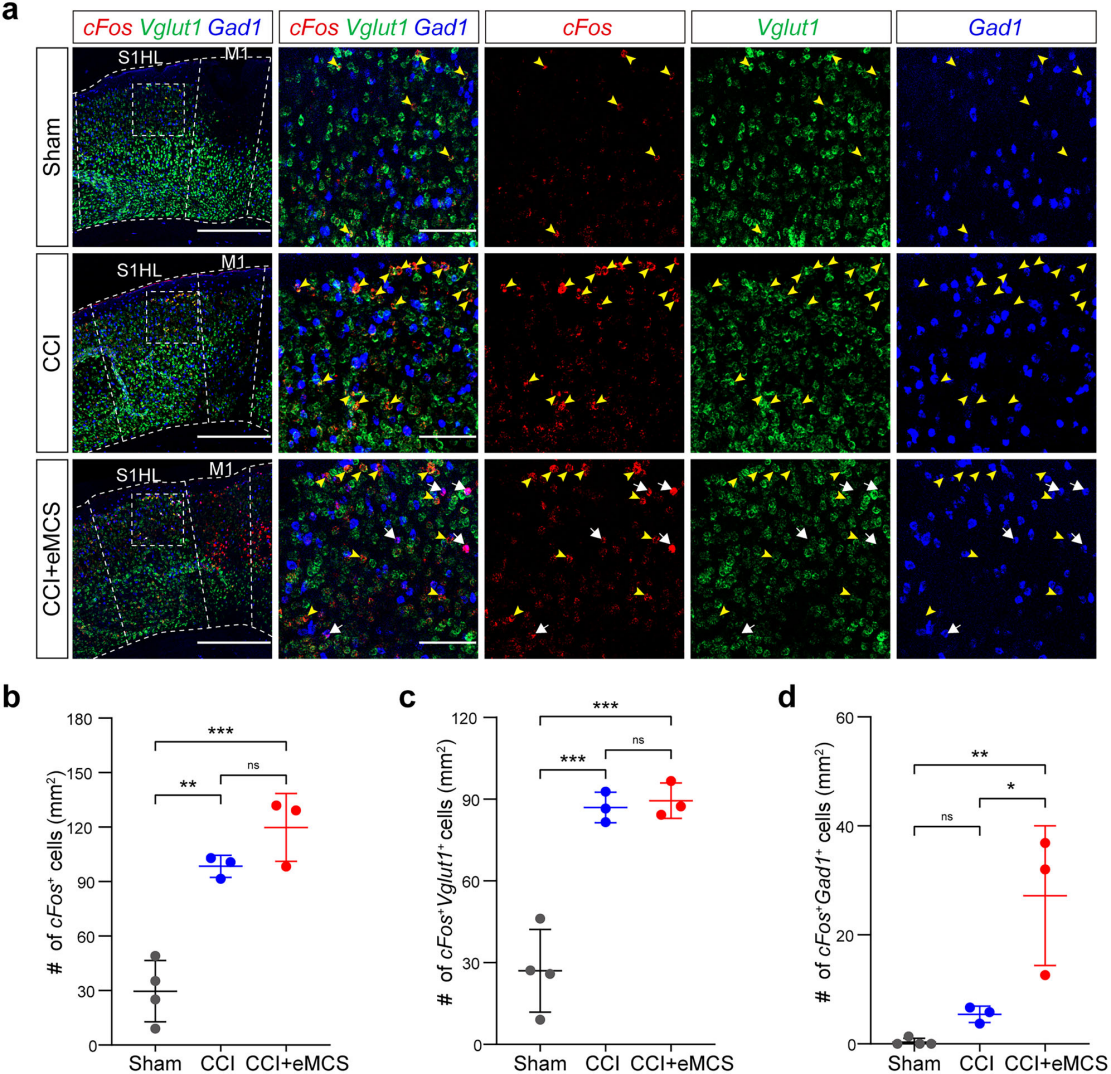
eMCS induces inhibitory interneurons activation in the S1. ***a***, Representative images of multiplex RNAscope in situ hybridization. Red, *c-fos*; green, *Vglut1*; blue, *Gad1*. In the left image, M1 and hindlimb S1 area (S1HL) are demarcated with white dashed lines. White dashed boxes indicate the regions magnified on the right. Sham, skin incision only; CCI, CCI surgery; CCI + eMCS, eMCS with CCI surgery. From top to bottom: sham, CCI, CCI + eMCS groups. White arrows, *c-fos*^+^ *Gad1*^+^ cells; yellow arrowheads, *c-fos*^+^ *Vglut1*^+^ cells. Scale bars: 500 µm (left column), 100 µm (right columns). ***b–d***, Quantification of the number of *c-fos*^+^ cells (***b***), *c-fos*^+^ *Vglut1*^+^ cells (***c***), and *c-fos*^+^ *Gad1*^+^ cells (***d***) in the S1HL. Sham group (*n* = 4); CCI group (*n* = 3); CCI + eMCS group (*n* = 3). Lines indicate the mean ± SD. ns, not significant; *p < 0.05, **p < 0.01, ***p < 0.001; one-way ANOVA followed by Tukey's test.

Given the role of the S1 in chronic pain regulation, we investigated whether eMCS alters neuronal activity in the S1, unlike chopMCS, which had no effect on the number of activated neurons in the S1 (Gan et al., 2022). To explore this, we conducted multiplex RNAscope experiments. Consistent with previous report (Cichon et al., 2017; Okada et al., 2021; Ding et al., 2023), the CCI group exhibited a significantly higher number of activated excitatory neurons (*c-fos*^+^ *Vglut1*^+^) in the S1 compared with the sham group [Fig. 3*a–c*; *c-fos*^+^ *Vglut1*^+^ cells (mean ± SD): sham, 27.03 ± 15.15; CCI, 86.94 ± 5.63]. However, there was no significant difference in the number of activated cells (*c-fos*^+^) and excitatory neurons (*c-fos*^+^ *Vglut1*^+^) between the CCI and CCI + eMCS groups, similar to what was observed with chopMCS [Gan et al., 2022; Fig. 3*a–c*; *c-fos*^+^ cells (mean ± SD): CCI, 98.41 ± 6.06; CCI + eMCS, 119.8 ± 18.74; *c-fos*^+^ *Vglut1*^+^ cells (mean ± SD): CCI, 86.94 ± 5.63; CCI + eMCS, 89.42 ± 6.49]. Notably, within the *c-fos*^+^ cells, inhibitory interneurons (*Gad1*^+^) were significantly more abundant in the CCI + eMCS group compared with both the sham and CCI groups (Fig. 3*a*,*d*; mean ± SD: sham, 0.34 ± 0.68; CCI, 5.42 ± 1.52; CCI + eMCS, 27.18 ± 12.83).

### eMCS differentially induces the activity of neurons in the S1

Neuropathic pain mouse models exhibit altered neuronal activity patterns in the S1, leading to increased spontaneous activity of LV-PNs. This increased activity of LV-PNs arises from a shift in neuronal circuits in the S1, where the activity of SST^+^ INs, which normally inhibit LV-PNs, is reduced due to increased suppression from VIP^+^ INs (Cichon et al., 2017; Ding et al., 2023). Importantly, restoring SST^+^ INs activity effectively suppresses pain responses, indicating that SST^+^ INs activity in the S1 plays a causal role in pain modulation.

**Figure 4.**
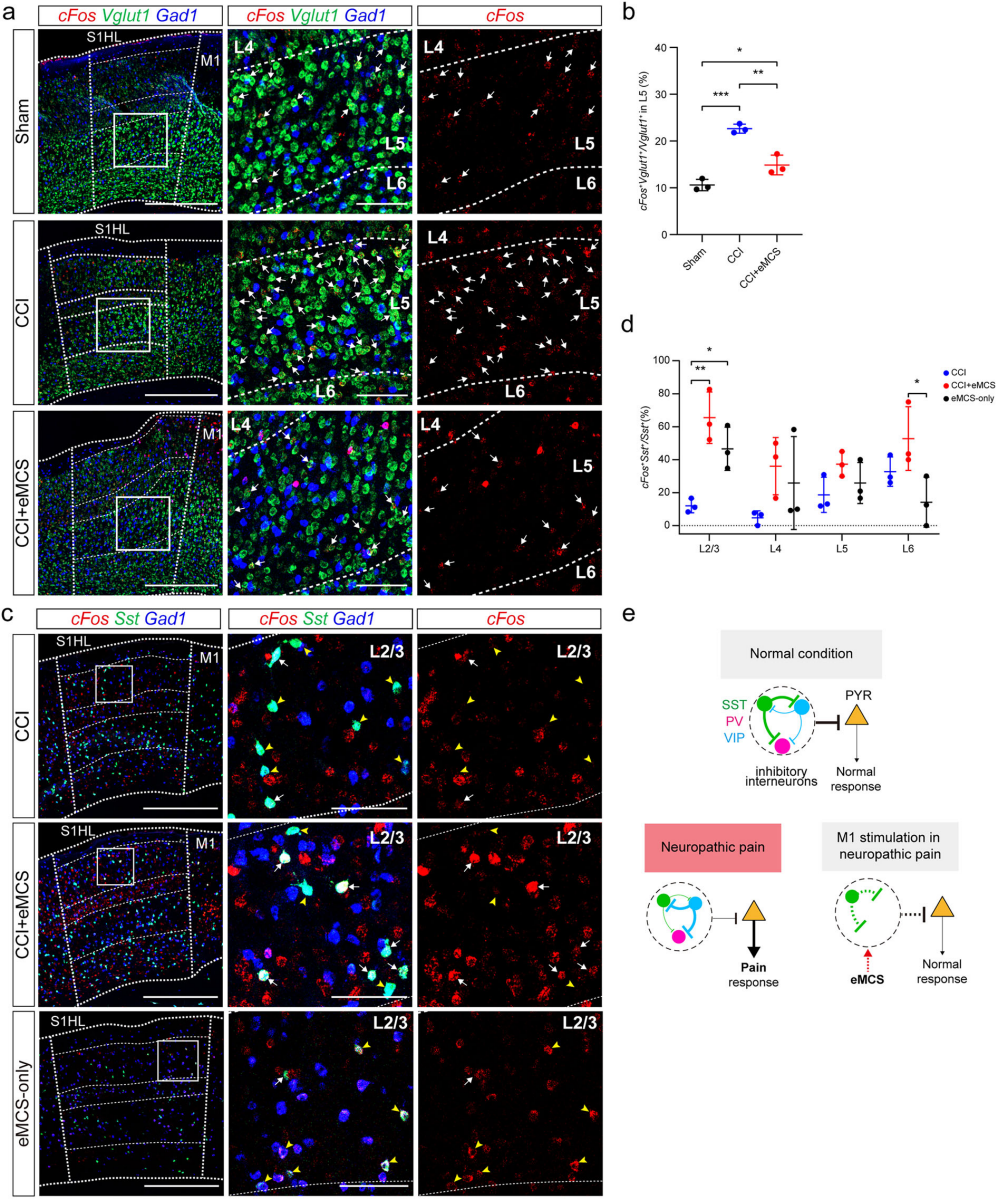
SST^+^ INs are highly activated by eMCS. ***a***, ***c***, Representative images of multiplex RNAscope in situ hybridization. Left, Layers in the hindlimb S1 area (S1HL) are demarcated with white dashed lines (from top to bottom: L1, L2/3, L4, L5, and L6). White boxes indicate the regions magnified on the right. Sham, skin incision only; CCI, CCI surgery; eMCS, CCI + eMCS with CCI surgery; eMCS-only, eMSC without CCI surgery. ***a***, Red, *c-fos*; green, *Vglut1*; blue, *Gad1*. White arrows, *c-fos*^+^ *Vglut1*^+^ cells. ***c***, Red, *c-fos*; green, *Sst*; blue, *Gad1*. White arrows, *c-fos*^+^ *Sst*^+^ cells; yellow arrowheads, *Sst*^+^ cells. Scale bars: 500 µm (left column), 100 µm (right columns). ***b***, Quantification of *c-fos*^+^ *Vglut1*^+^ cells in layer V of the S1HL. Sham group (*n* = 3); CCI group (*n* = 3); CCI + eMCS group (*n* = 3). Lines represent the mean ± SD. *p < 0.05; **p < 0.01; ***p < 0.001; one-way ANOVA followed by Tukey's test. ***d***, Quantification of *c-fos*^+^ *Sst*^+^ cells in each layer of the S1HL. CCI group (*n* = 3); CCI + eMCS group (*n* = 3); eMCS-only group (*n* = 3). Lines represent the mean ± SD. *p < 0.05; **p < 0.01; one-way ANOVA followed by Tukey's test. ***e***, S1HL local circuits under normal condition (top), neuropathic pain condition (CCI, bottom left), and neuropathic pain condition with eMCS treatment (CCI + eMCS, bottom right). Thickness of lines denotes the strength of the signals; dashed lines, predicted changes in the strength of the signals based on ***a*** and ***c***. Green circles, SST^+^ INs; purple circles, parvalbumin-positive interneurons (PV^+^ INs); blue circles, VIP^+^ INs; yellow triangles, LV-PNs. This model is modified from the previous result (Cichon et al., 2017). See also Extended Data Figure 4-1.

To investigate potential differential responses to eMCS among S1 neurons, we performed multiplex RNAscope experiments including interneuron subtype markers. In the CCI group, the number of activated LV-PNs (*c-fos*^+^ *Vglut1*^+^) was larger than in the sham group, while it was significantly reduced in the CCI + eMCS group compared with the CCI group (Fig. 4*a*,*b*; mean ± SD: sham, 10.61 ± 1.22; CCI, 22.65 ± 0.98; CCI + eMCS, 14.87 ± 2.10). To investigate how the number of activated LV-PNs was reduced in the CCI + eMCS group, we first examined the proportion of activated inhibitory interneurons in the S1. While the proportion of SST^+^ INs (*Sst*^+^/*Gad1*^+^) in inhibitory interneurons remained unchanged, the number of activated SST^+^ INs (*c-fos*^+^ *Sst*^+^/*Sst*^+^) significantly increased in the CCI + eMCS group compared with the CCI group [Fig. 4*c*, Extended Data Fig. 4-1*a*; *Sst*^+^/*Gad1*^+^ (mean ± SD): CCI, 29.18 ± 3.17; CCI + eMCS, 32.04 ± 1.72; *c-fos*^+^ *Sst*^+^/*Gad1*^+^ (mean ± SD): CCI, 5.38 ± 0.80; CCI + eMCS, 15.59 ± 0.56]. Furthermore, the proportion of activated SST^+^ INs (*c-fos*^+^ *Sst*^+^/*Gad1*^+^) among inhibitory interneurons was positively correlated with the analgesic effects observed during eMCS (Extended Data Fig. 4-1*b*). Subsequently, we further examined the changes in non-SST INs (*Gad1* *^+^* *Sst^−^*). The number of activated non-SST INs (*c-fos*^+^ *Gad1^+^ Sst^−^*) was not significantly different between groups (Fig. 4*c*, Extended Data Fig. 4-1*d*; mean ± SD: CCI, 15.08 ± 9.50; CCI + eMCS, 24.42 ± 7.32). The increased number of activated SST*^+^* INs were enriched in layer 2/3 of the S1 [Fig. 4*d*, Extended Data Fig. 4-1*e–f*; *c-fos*^+^ *Sst*^+^/*Sst*^+^ (%) in layer 2/3 (mean ± SD): CCI, 12.04 ± 4.24; CCI + eMCS, 65.44 ± 15.59; eMCS-only, 46.62 ± 13.08], suggesting an interaction with the apical dendrites of LV-PNs in this layer. These data suggest that eMCS inhibits the heightened activity of LV-PNs in neuropathic pain mouse models by specifically activating S1 SST*^+^* INs (Fig. 4*e*), although other mechanisms may also be involved.

10.1523/ENEURO.0541-24.2025.f4-1 Figure 4-1 **Layer-specific changes in neuronal activity induced by eMCS in the S1HL.** (**a**) Quantification of *Sst*^+^ cells in the S1HL. CCI group (n=3); CCI + eMCS group (n=3). Lines indicate the mean ± SD. Only statistically significant differences are indicated: ****p < 0.0001; unpaired t-test. (**b**) Simple linear regression analysis between the percentage (%) of *c-Fos*^+^*Sst*^+^ cells / *Gad1*^+^ cells and the pain response (AUC of von Frey assay during eMCS). (**c**, **d**) Quantification of *Gad1*^+^ cells in the S1HL. The number of *c-Fos*^+^*Gad1*^+^ cells (**c**) and *c-Fos*^+^*Gad1*^+^*Sst*^-^ cells (d) in the S1HL. Lines indicate the mean ± SD. Only statistically significant differences are indicated: *p < 0.05; unpaired t-test. (**e**–**g**) Distribution of *c-Fos*^+^*Gad1*^+^ cells (**e**), *Gad1*^+^ cells (**f**), and *Sst*^+^ cells (**g**) across the layers of the S1HL. Lines indicate the mean ± SD. Only statistically significant differences are indicated: *p < 0.05; unpaired t-test. Download Figure 4-1, TIF file.

To determine whether the activation of S1 SST*^+^* INs by eMCS occurs exclusively under pain conditions, we performed eMCS in animals without CCI surgery. We observed that eMCS increased the number of *c-fos^+^* cells in layer 2/3 of S1, similar to the increase observed in the CCI group receiving eMCS [Fig. 4*c*,*d*; mean ± SD: *c-fos*^+^ *Sst*^+^/*Sst*^+^ (%) in layer 2/3 (mean ± SD): CCI, 12.04 ± 4.24; CCI + eMCS, 65.44 ± 15.59; eMCS-only, 46.62 ± 13.08]. To investigate whether eMCS activates S1 SST*^+^* INs through circuit mechanisms or physical proximity between brain regions, we examined *c-fos*^+^ cells in brain areas adjacent to M1, including M2 and ACC. Although the overall number of *c-fos*^+^ cells in the M2 was increased in the CCI + eMCS group, the number of *c-fos*^+^ cells specifically in layer 2/3 was not significantly different across groups (Extended Data Fig. 2-2*e*; mean ± SD: sham, 0.77 ± 0.52; CCI, 1.18 ± 1.37; CCI + eMCS, 3.80 ± 2.49).

## Discussion

Stimulation of the M1 has been widely tested for the treatment of chronic pain conditions. In a recent paper, the analgesic effects of chopMCS were found to be mediated by excitatory neurons in the ZI and PAG that receive excitatory inputs from LV-PNs of the hindlimb area of the M1 (Gan et al., 2022). While the paper reported no changes in the number of activated neurons in the S1 following chopMCS, a growing body of evidence suggests the S1 plays a crucial role in chronic pain. This is evidenced by studies demonstrating altered neuronal activities within the S1 under chronic pain conditions, where manipulating such activity has shown potential for alleviating pain responses (Cichon et al., 2017; Du et al., 2017; Liu et al., 2018; Wei et al., 2021; Kim and Kim, 2022; Ding et al., 2023). Our findings corroborate previous studies highlighting the role of the S1 in chronic pain responses. We observed significantly higher activation of SST^+^ INs compared with non-SST INs following eMCS. This suggests that SST^+^ INs may inhibit LV-PNs activity in the S1, potentially contributing to reduction in pain responses. While chopMCS did not alter the overall number of activated neurons in the S1, our study revealed changes within this region by examining differential responses among neuronal subtypes. This discrepancy between chopMCS and eMCS could be attributed to inherent differences in the stimulation methods employed or simply to the broader activation induced by eMCS compared with chopMCS, which cannot be ruled out. Furthermore, the optogenetic and chemogenetic stimulation used in chopMCS differ from the electrical stimulation employed in eMCS in terms of frequency, intensity of neuronal activation, and downstream signaling pathways. Importantly, future research should address the antidromic and orthodromic activation of neurons induced by eMCS.

In addition to inducing the differential response in the S1, eMCS induced a distinct temporal response, unnoticed in chopMCS. eMCS elicited a persistent analgesic effect that extended beyond the stimulation period, a phenomenon not observed in chopMCS. This prolonged analgesia may be mediated by plasticity changes within the S1. SST^+^ INs are known to play a crucial role in synaptic plasticity, with their activity being modulated by repetitive sensory stimulation and learning (Kato et al., 2015; Adler et al., 2019; Song et al., 2021). Dysregulation of these interneurons has been implicated in various neurological disorders. Given the sustained analgesic effects observed for at least 2 d after stimulation cessation and evidence that high-frequency stimulation of vibrissal M1 projection neurons facilitates SST^+^ INs activity in the barrel cortex (Lee et al., 2013), it is plausible that our high-frequency eMCS paradigm induced plasticity changes within the S1, particularly in SST^+^ INs. Identifying these plasticity changes could reveal targets for reversing chronic pain conditions and achieving long-lasting analgesic effects. Furthermore, this approach holds promise for treating a range of neurological disorders characterized by SST^+^ INs dysfunction.

Given the diverse cellular makeup of cortical areas (Zeisel et al., 2015; Di Bella et al., 2021) and the multifaceted nature of chronic pain, which originates from various causes and involves different sensory modalities (Colloca et al., 2017; Finnerup et al., 2021; Fiore et al., 2023), cellular and circuit-level changes within the cortex likely vary depending on the specific types of chronic pain. This is supported by findings demonstrating differential engagement of cortical pathways to the spinal cord allodynia induced by heat and cold versus mechanical stimuli, with only mechanical allodynia being suppressed by pyramidotomy (Atasoy et al., 2012; Liu et al., 2018). Considering that chopMCS suppressed both mechanical and cold-induced allodynia, eMCS may also be effective in regulating both mechanical allodynia (as demonstrated in this study) and cold-induced allodynia (to be explored in future studies) by modulating sensory circuits in the spinal cord through both the direct pathway to the spinal cord and the indirect pathways to the PAG and ZI. This suggests that different populations of pyramidal neurons in the S1 are involved in pain regulation in a sensory modality-dependent manner. Recent advancements in sequencing technologies have begun to unravel the underlying mechanisms of chronic pain (Wang et al., 2021; Jung et al., 2023), paving the way for systemic analyses in both model organisms and human subjects. By leveraging these sequencing technologies alongside genetic tools, future study can elucidate the molecular details of S1 changes associated with various chronic pain conditions and specific alterations induced by eMCS.

### Limitations of the study

In this paper, we identified increased activity of SST^+^ INs in the S1 upon eMCS. While our findings and previous studies suggest a potential role for SST^+^ INs in mediating the analgesic effects of eMCS, further investigation is warranted to establish a causal link. However, unraveling the contribution of SST^+^ INs in pain suppression is challenging, due to simultaneous activation of other pain-suppressing regions by eMCS. Furthermore, the electrophysiological properties of SST^+^ INs following each eMCS should be examined to determine whether plasticity changes occurred in the SST^+^ INs. Our experiments exclusively used male mice in a CCI model and assessed the analgesic effects of eMCS using the von Frey assay to test mechanical sensitivity. Future studies should expand these experimental parameters, including testing cold sensitivity, to determine the generalizability of our conclusions beyond these specific conditions.

## Data Availability

All data generated or analyzed during this study are included in this published article.

## Synthesis

Reviewing Editor: Jennifer Dulin, Texas A&M University Decisions are customarily a result of the Reviewing Editor and the peer reviewers coming together and discussing their recommendations until a consensus is reached. When revisions are invited, a fact-based synthesis statement explaining their decision and outlining what is needed to prepare a revision will be listed below. The following reviewer(s) agreed to reveal their identity: Yu Shin Kim.

The authors agree that the revised manuscript is much improved. However, there remain some lingering concerns regarding correlation vs. causation and the limitations of a small group size. All of the concerns should be addressed in a revised version of the manuscript. Please see point-by-point comments, below.

Reviewers' comments:

Reviewer #1:

The authors have made acceptable revisions to the manuscript. The primary concerns with this study are 1) the lack of causality and 2) the low sample size. However, that is acceptable with the addition of speculative statements removed from the Results and more cautious conclusions in the Discussion.

Lines 269-271: This is speculation and should be in the Discussion rather than the Results.

Lines 273-274: To say: "...indicating increased spontaneous activity of pyramidal neurons", you must state that the Fos+ neurons are pyramidal in nature or have characteristics of being pyramidal (i.e., vGlut1-expressing).

Lines 280-286: The authors cannot state ZI and PAG "exhibited an increased number of c-Fos+ cells compared to both the sham and pain groups" and follow up by stating that the "increase" observed in ZI did not reach statistical significance compared to the pain group. If it was not significantly different, you cannot claim there was an increase attributable to eMCS, regardless of previous findings.

Lines 327-329: This is speculation and should be in the Discussion rather than the Results.

Reviewer #2:

This study explores the involvement of the primary somatosensory cortex (S1) in the analgesic effects elicited by electrical stimulation of the primary motor cortex (M1), termed eMCS, within a neuropathic pain mouse model induced via chronic constriction injury (CCI). This work builds upon prior findings by Gan et al. (2022, Science), which dissected layer-specific projections from M1, revealing distinct pathways for modulating sensory hypersensitivity (via layer 5 to ZI and PAG) and affective components of pain (via layer 6 to mediodorsal thalamus). Compared to the Gan et al. study, which used sophisticated optogenetic and chemogenetic tools to dissect projection-specific circuits, the present study takes a complementary but distinct approach, demonstrating that eMCS can increases the activity of inhibitory interneurons in S1, and particularly activating S1 SST⁺ interneurons and suppressing LV-PN hyperactivity associated with neuropathic pain. However, as acknowledged by the reviewers and the authors themselves, the study remains correlative. While causality remains unproven, the authors have addressed reviewer concerns thoughtfully and provided new data that strengthen their claims.

Major concerns:

(1) Cold allodynia is a hallmark feature of many neuropathic pain conditions and is often used alongside mechanical hypersensitivity to provide a comprehensive assessment of pain sensitivity. The current study relies solely on the von Frey assay to evaluate mechanical thresholds, but does not address cold sensitivity, which limits the scope of the analgesic evaluation. If cold sensitivity was not assessed, the authors should explicitly acknowledge this in the limitations section, and discuss how inclusion of this assay in future studies would provide a more comprehensive understanding of eMCS-mediated analgesia.

(2) The behavioral data in this study are based on n = 3 animals per condition, which raises concerns regarding statistical power and reproducibility. Although the authors report significant differences and consistent trends, the small sample size limits the generalizability of the findings and increases susceptibility to outliers. Could the authors clarify whether the low n is due to technical limitations, such as difficulties with electrode implantation, post-surgical recovery, or variability in pain model induction? Were there significant animal losses or exclusions based on surgical complications or data quality?

(3) To improve clarity and avoid confusion, I recommend reformatting the figure labels throughout the manuscript to consistently reflect the experimental groups as "Sham," "CCI," and "CCI + eMCS." This terminology aligns better with the study design and will help readers more easily interpret the results. Additionally, data from the eMCS-only group (without CCI) should be included in each relevant figure. While the authors have included histological data from the eMCS-only group (without CCI) to examine neuronal activation, it is concerning that behavioral data from this group are still missing. Including eMCS-only behavioral data is essential to determine whether electrical stimulation alone alters pain sensitivity or baseline behavior in the absence of nerve injury.

(4) The manuscript does not clearly specify on which day(s) c-Fos expression was assessed in the eMCS protocol and behavioral timeline. Based on the statement, "Tissues from the same mice used to measure withdrawal thresholds were collected for histological analysis," I infer that animals were sacrificed after the 30-day behavioral testing period, during which pain sensitivity had returned to baseline for at least three days. However, the behavioral data indicate that eMCS-induced analgesia begins to emerge on Day 17, whereas Day 16 (the first day of stimulation) shows no significant change in withdrawal thresholds. If c-Fos analysis was performed on or immediately after Day 16, it may reflect early neuronal activation rather than activity during the peak analgesic effect. This raises a critical concern: the c-Fos data may not correspond to the period of effective pain relief, and therefore may not reflect the neuronal correlates of analgesia.

Minor concerns:

The title for Fig.3. should be revised to "eMCS induces inhibitory interneurons activation in the S1".

## Author Response

Comments from the Editors Synthesis of Reviews:

Synthesis Statement for Author (Required):

While the reviewers agree that this study addresses an important topic and the manuscript is well written, some major concerns preclude publication in eNeuro in the manuscript's current form. Chiefly, both reviewers agreed that the results were correlative and that additional experiments would be needed to determine whether the change in interneuron recruitment is related to amelioration of pain, rather than a general phenomenon that follows from orthodromic recruitment of feedforward inhibition. Please see below for a point-by-point critique of the manuscript.

Reviewers' comments:

Reviewer #1:

Manipulation of motor cortex through electrical microstimulation or other methods can alleviate symptoms of chronic pain. Previous studies have implicated subcortical regions including PAG and ZI, which are targets of extratelenchephalic pyramidal neurons in motor cortex, in this effect. The authors of this study hypothesize that somatosensory cortex is also involved. To test this hypothesis the authors microstimulate motor cortex and use in situ hybridization to measure changes in c-fos expression in excitatory and inhibitory neurons in S1. The key finding was that microstimulation of motor cortex causes a disproportionate increase in SST interneuron fos expression in S1. The authors point out this suggests microstimulation leads to the suppression of cortical hyperactivity that follows from injury-induced pain. These findings are interesting, but I have some major concerns that center around the general approach. The way the experiment was constructed, it is not possible to determine if the change in interneuron recruitment just a general feature of feedforward inhibition caused by orthodromic stimulation, or if it is causally related to the amelioration of pain. Perhaps one way to tease apart this question would be count fos activity in the days following eMCS, where there is still a clear analgesic effect. More fundamentally, the authors could/should perform eMCS in sham animals. If there is no change in SST neuron recruitment in sham animals, the conclusions would be supported. However, in the case there were still changes, it would not reject their hypothesis but require follow-up analysis and/or experiments. There are more complicated and direct experiments that could also be performed (chemogenetic SST inactivation in S1 during microstimulation), but they are perhaps outside this scope.

As suggested by Reviewer #1, we conducted additional experiments to perform eMCS without concurrent CCI surgery and analyzed the activation of c-Fos+cells in S1, as shown in Fig. 4-2. Our data clearly demonstrate that eMCS alone effectively activates Sst+ neurons in S1, supporting our hypothesis that eMCS reactivates these neurons, which are otherwise inhibited under chronic pain conditions. Although one might initially interpret this finding as evidence that eMCS simply activates Sst+neurons through direct circuit mechanisms, the sustained pain-suppressive effects observed for a couple of days after cessation of eMCS strongly suggest that more enduring plasticity changes likely occur within pain-suppression circuits.

To further address potential concerns regarding the specificity of our finding, we quantified c-Fos+cells in cortical regions adjacent to M1, including M2 and ACC, as shown in Fig. 2-2. These additional analyses indicate that physical proximity alone does not account for the observed activation of Sst+ neurons in S1 following eMCS. Thus, we believe our data support a targeted and selective circuit-level mechanism rather than a nonspecific spatial effect.

Important questions remain for future research, including elucidating the precise mechanisms underlying eMCS-mediated activation of Sst+ neurons in S1, characterizing the specific neuronal changes induced by chronic pain conditions, and determining how eMCS produces sustained analgesic effects.

We appreciate the reviewer's insightful comments, which have significantly strengthened our manuscript.

Detailed responses addressing each of the reviewer's comments and suggestions are provided below.

I have some other comments and suggestions:

1. While previous literature may demonstrate a mechanism acting though projection neurons in layer 5, I do not see any evidence for such a claim (i.e., lines 60, 78, and beyond) in the data presented here. The authors should either: 1) provide clear evidence that electrical stimulation is restricted to layer 5 (histological, current sink, genetic, viral, or otherwise), or 2) be more agnostic about the circuit mechanism of their results. The authors already have measurements of c-fos expression following eMCS, so this could simply be a depth analysis of expression. However, I suspect that the layers stimulated are broader, based on the in situs in Figure 2A. Indeed, the statement in line 109 should be justified with data.

We acknowledge Reviewer #1's concern regarding the specificity of stimulation targeting layer 5. Due to methodological limitations, we cannot fully address the issue of targeting specificity. However, as shown in Fig. 2-2, an indirect method reveals that the majority of c-Fos+cells are located in layer 5. The coordinates of electrode placement were determined using established reference coordinates, such as the Allen Brain Atlas (https://mouse.brain-map.org/static/atlas) and the book by Paxinos, G., &Franklin, K. B. (2019).

Additionally, in the revised version of the Discussion section, we have added a cautionary note regarding data interpretation as follows: " This discrepancy between chopMCS and eMCS could be attributed to inherent differences in the stimulation methods employed or simply to the broader activation induced by eMCS compared to chopMCS, which cannot be ruled out." 2. The Methods section does not provide enough detail on experimental procedures. For instance, the depth of electrode placement should be indicated in the Electrode Implantation section.

As suggested by Reviewer #1, we have included the details of electrode placement in the Materials and Methods section (Electrode Implantation) as follows: "...multi-channel silicon probe (A4x4-3mm-100-125-703-CM16LP, NeuroNexus) on the hindlimb motor cortex (1.0 mm rostral and 1.0 mm lateral to the bregma at a depth of 0.9 mm from the cortical surface)..." Additionally, detailed protocols have been incorporated as Reviewer #1 suggested in other comments (Comment 3, 4, 5, 6, and 15).

3. In addition, the protocol for setting the intensity of electrical stimulation (lines 84-85, 258-260) is unclear. The authors sought to limit "obvious behavioral responses" or interference "with the animals' general behaviors", but what exactly does this mean? For example, did electrical stimulation cause any movements? If the authors have behavioral quantification (ideal), they should present it.

As suggested by Reviewer #1, we have included behavioral quantification data, as shown in Fig. 1-1. In this analysis, we tracked animal movements before and during eMCS (without CCI surgery) using idtracker.ai, an AI-based animal tracking software. The position of the animal in the cage was analyzed, and no differences were observed in overall speed, travel distance, or the proportion of resting time before and during eMCS.

The detailed protocol has been added to the Materials and Methods section (Behavioral analysis) as follows: "Animal movements were tracked using idtracker.ai (5.2.12) (Romero-Ferrero et al, 2019). Movement parameters, including..." 4. Is there a reason AUC used in Fig 1C? There is a more direct measurement (threshold) available for plotting.

In the original analysis, we measured AUC across multiple days during the von Frey assay to incorporate data over an extended period for comparison. However, following the suggestion by Reviewer #1, we have revised our analysis and directly compared withdrawal responses between groups on specific individual days: Day 5 and Day 13 (pre-eMCS), Day 19 (eMCS), and Day 23 and Day 29 (post-eMCS).

We have revised Fig. 1c accordingly and performed one-way ANOVA followed by Tukey's test. The detailed statistical analysis data can be found in Fig. 1-2 5. Were in situ hybridization experiments performed in separate animals or in the same animals as in Figure 1? I presume they are the same mice, but that is not clear in the Methods. It is possible that plasticity (which the authors posit as a potential consequence of their stimulus protocol) means interneuron recruitment would be different at the beginning of eMCS versus the end of the protocol. The authors should detail their methods and comment on the implications.

We agree with the Reviewer #1's concern. In the revised version of the Materials and Methods section (RNA in situ hybridization), we have explicitly stated that the same mice were used for both behavioral and histological analyses, as follows: " Tissues from the same mice used to measure withdrawal thresholds were collected for histological analysis...." As suggested by Reviewer #1, the recruitment of interneurons may differ during eMCS. In the revised version, we performed eMCS (without CCI surgery) stimulation for 2 days and quantified c-Fos expression in S1, as now shown in Fig. 4-2. Similar to eMCS (with CCI surgery), the number of c-Fos and Sst double-positive cells in layer 2/3 of S1 was greater than in the Sham (incision-only) group. This suggests that the activation of Sst neurons in S1 by eMCS may result from an acute effect of eMCS; however, the potential contribution of plasticity cannot be ruled out since the analgesic effect persists for couple of days following the cessation of eMCS.

6. The authors must add detailed information for how c-fos expression was quantified. What a thresholding approach used? Were neurons counted manually using blinding? These are particularly important where the signal is graded and often faint, as it appears in Figure 2. Same for Figure 3.

We agree with Reviewer #1's concern. In the revised version of the Materials and Methods (Quantification of c-Fox expression), we have provided a detailed description of the quantification process, which read as follows: " c-Fos expression was quantified manually under blinded conditions. All slides were stained under identical experimental conditions and imaged using consistent confocal settings for the same probe sets. The images were then processed in Fiji. c-Fos expression was counted when the signal overlapped by more than 50% with DAPI, Vglut1, Gad1, Sst, or Vglut2 signals...." 7. I do not understand why there is no c-fos expression in the spinal cord. While the authors claim this is evidence that analgesic effects of eMCS are mediated supraspinally, would one not expect to see spinal c-fos expression through ongoing spinal activity? Was electrical stimulation for c-fos experiments performed under anesthesia, in which case I would expect less fos activity in the spinal cord? Or is the in situ protocol not optimized for spinal tissue? We acknowledge that the chromogenic detection method for in situ hybridization is less sensitive compared to fluorescence-based methods. To address this limitation, we have applied multiplex RNAscope in situ hybridization, as now have shown in Fig. 2-1. Using this approach, we quantified and compared the number of c-Fos+cells in the dorsal horn across groups. Although the number of c-Fos+cells in the eMCS was the largest, the overall number of c-Fos+cells was low, and there was no statistical difference between the pain and eMCS groups.

Typically, peripheral stimuli are used to induce neuronal activation when comparing responses in the dorsal horn, as reported by Liu, Yuanyuan, et al. (Nature, 2018). However, in our experiment, we stimulated M1 without applying peripheral stimuli, which may account for the low number of c-Fos+cells observed in the dorsal horn of the CCI model mice.

8. Lines 114-116: couldn't this effect be due to increased excitability to electrical stimulation, or some other mechanism instead of increased spontaneous activity? The text referred to by Reviewer #1 discusses the changes in the number of c-Fos+cells between the sham and pain groups. Consistent with previous research, the number of c c-Fos+cells was greater in the pain group compared to the sham group.

As Reviewer #1 pointed out, eMCS can influence the activity of neurons in the cortex including layer 5 neurons in S1.

9. Quantification of c-fos expression in motor cortex should be presented for sham, pain, and eMCS.

As suggested by Reviewer #1, we have compared the number of c-Fos+cells in M1, as now shown in Fig. 2-2. Our analysis revealed that the number of c-Fos+cells in layer V of the M1 is the largest among all groups, supporting preferential activation of layer V. Interestingly, even in the eMCS group, the c-Fos expression patterns in nearby brain regions, such as M2 and ACC, differ. This suggests that the effect of eMCS is less likely due to physical proximity and is instead mediated through neuronal circuits within the cortex.

10. Electrical stimulation can evoke both orthodromic and antidromic responses. The authors should comment in the Discussion how they cannot identify either as the cause for their results. I also worry that their electrical stimulation protocol could directly activate neighboring (i.e., somatosensory) cortical regions. The authors should compare their stimulation protocol to existing protocols that demonstrate this is or is not the case. They could potentially also count neurons in other nearby cortical regions, such as cingulate. If there is regional specificity in eMCS-induced cell type activity, this would support their conclusions.

We acknowledge Reviewer #1's concerns regarding the mechanisms underlying activation of S1 by eMCS. To rule out the potential confounding effect of physical proximity, we quantified the number of c-Fos+cells across cortical layers in adjacent brain regions, as shown in Fig. 2-2. Unlike in S1, the number of c-Fos+cells in Layer 2/3 of M2 and ACC did not significantly differ from that observed in the sham and pain groups. These results suggest that physical proximity alone is unlikely to be the primary cause of signal transmission following eMCS; instead, activation is likely mediated through specific neuronal circuits within the cortex.

Furthermore, in the revised version of the Discussion section, we have included a cautionary comment addressing the directionality of neuronal activation induced by eMCS, as follows: "Importantly, future research should address the antidromic and orthodromic activation of neurons induced by eMCS." 11. According to the data in Figure 2F (n.s.), eMCS does not lead to a significant increase in fos expression in ZI compared to the pain group, contrary to the claim in line 118.

Thank you for highlighting this issue. In the revised version, we have corrected the statement in the Result section as follows: "While the increase in c-Fos+cells within the PAG following eMCS was statistically significant compared to both the sham and pain groups, the increase observed in the ZI following eMCS did not reach statistical significance compared to the pain group, although the average number of c-Fos+cells was higher." 12. I do not think VIP neurons should be included in the cartoons in Figure 4E, since there is no data in this study to suggest they are involved.

We agree with Reviewer #1's comment on Fig. 4e. As suggested, we have removed VIP and PV neurons from the eMCS portion of Fig. 4e in the revised version.

13. First citation (Towne, C. et al. 2009) does not make sense in its context.

We have removed the citation as suggested by Reviewer #1 and have thoroughly verified that all references are properly included.

14. Three animals per condition is a pretty small sample size for quantification of a relatively noisy signal.

We acknowledge that noisy signals are common concern in biological experiments. To minimize variability at each step, we controlled the experimental setups as follows: we used only male mice at similar ages (8-10 weeks old) and refined our technique to efficiently generate the CCI model. In our hands, the generation of the CCI model was robust, and the pain-suppression effect of eMCS was consistent. As a result, the behavioral and histological results were statistically significant even with only three animals per condition.

15. How were different brain regions identified? For defining brain regions, we referred to the Allen Brain Atlas (https://mouse.brain-map.org/static/atlas) and the book by Paxinos, G., &Franklin, K. B. (2019). For defining Rexed laminae in the spinal cord, we referred to the book by G. Sengul (2013). These resources are well-established and widely used for anatomical delineation in neuroscience research. Additionally, in our histological experiments, we included DAPI signals to identify cell density, which serves as a key landmark for defining brain regions and layers.

We have cited these references in the materials and methods section (Quantification of c-Fos expression) as follows: "Brain regions and cortical layers, as well as Rexed laminae in the dorsal horn of the spinal cord, were defined according to established reference atlases (Paxinos et al, 2019; Allen Institute for Brain Science, 2011)." Reviewer #2:

Overview:

The authors felt analysis of subpopulations of neurons in primary somatosensory cortex (S1) would be informative about analgesic mechanisms of electrical primary motor cortex (M1) stimulation (eMCS). To this end, they implanted electrodes into layer 5 of M1 to perform eMCS on mice treated with chronic constriction injury (CCI). eMCS decreased mechanical pain sensitivity in CCI mice and sensitivity did not return to baseline until 2 days after eMCS ceased. The authors measured neuron activation by staining for c-fos mRNA. C-fos-positive cells increased periaqueductal gray and zona inserta. Furthermore, c-fos was increased in interneurons in the S1, decreased in layer 5 S1 excitatory neurons, and increased in layer 2/3 S1 somatostatin-positive interneurons. The authors list substantial differences between eMCS and activation using chemogenetics and optogenetics, indicating those methods are an imperfect model of eMCS in humans.

These results are consistent with prior literature. The study is, as the authors note, correlative and do not establish causation.

Strengths:

The authors establish the eMCS mouse model and show that chemogenetics and optogenetics are imperfect substitutes for eMCS. The limitations section does a good job of covering some major limitations.

Weaknesses:

There are substantial weaknesses which were not addressed, or not directly addressed, by the limitations section. One weakness is the lack of different pain modalities tested. There were not tests for cold or thermal pain, grimace score, or other means of assessing pain (which the authors alluded to in the Limitations section)). They over rely on von Frey. One method of data analysis is poorly explained. Another major weakness is the sample sizes are minimal. However, this weakness is somewhat mitigated by the fact that the results are consistent with what is found in other literature and that the ranges are often so far apart that the data still look convincing.

Required changes:

The authors need to describe the normalization to AUC. It is unclear where the normalization starts (I assume day 1) or ends (I assume the final day). Based on the graph numbers, I think they are trying to say they Integrated area under the curve and divided by the number of trials for the area integrated, or something similar, but this is unclear.

In the original analysis, we measured AUC across multiple days during the von Frey assay to incorporate data over an extended period for comparison. However, following the suggestion by Reviewer #1, we have revised our analysis and directly compared withdrawal responses between groups on specific individual days: Day 5 and Day 13 (pre-eMCS), Day 19 (eMCS), and Day 23 and Day 29 (post-eMCS).

We have revised Fig. 1c accordingly and performed one-way ANOVA followed by Tukey's test. The detailed statistical analysis data can be found in Fig. 1-2 As suggested by Reviewer #2, we have added dashed lines to demarcate layer 5 in Fig. 2a and Fig. 2-2a.

Recommendation This is not a broad, sweeping study. It seems like a set up for future studies or publications. Overall, with some minor changes, the study may be published.
