# Supplementary material for: Electrical Stimulation of the M1 Activates Somatostatin Interneurons in the S1: Potential Mechanisms Underlying Pain Suppression
Source: eNeuro. 2025 Apr 25;12(4):ENEURO.0541-24.2025. doi: 10.1523/ENEURO.0541-24.2025 (PMC12043047; doi:10.1523/ENEURO.0541-24.2025)
Supplement: Figure 1-1 — Two-way ANOVA test of von Frey assay. Mechanical sensitivity determined by force required for 50% threshold for paw withdrawal, as determined through von Frey assay. eMCS was performed from Day 16 to Day 22. Sham: skin incision only; CCI: CCI surgery; CCI + eMCS: eMCS with CCI surgery. Sham group (n = 4); CCI group (n = 3); CCI + eMCS group (n = 3). A statistical test was performed between groups on each day post-surgery: ns, not significant; *p < 0.05; **p < 0.01; ***p < 0.001; ****p < 0.0001; two-way ANOVA followed by Tukey's test. Download Figure 1-1, DOC file. [file eneuro-12-ENEURO.0541-24.2025-s002.doc]

**Fig. 1-2**

| Day | eMCS vs Sham | | Pain vs Sham | | eMCS vs Pain | |
| --- | --- | --- | --- | --- | --- | --- |
|  | p-value | | p-value | | p-value | |
| 1 | 0.2658 | ns | 0.5125 | ns | 0.0362 | * |
| 3 | 0.7415 | ns | > 0.9999 | ns | 0.7759 | ns |
| 5 | 0.6098 | ns | 0.4364 | ns | 0.9624 | ns |
| 7 | 0.7519 | ns | 0.7552 | ns | > 0.9999 | ns |
| 9 | 0.6269 | ns | 0.9252 | ns | 0.4465 | ns |
| 11 | 0.1459 | ns | 0.0212 | * | 0.7310 | ns |
| 13 | 0.0123 | * | 0.0240 | * | 0.9733 | ns |
| 14 | 0.0447 | * | 0.0355 | * | 0.9961 | ns |
| 15 | 0.0100 | * | 0.0133 | * | 0.9957 | ns |
| 16 | 0.1868 | ns | 0.0084 | ** | 0.4719 | ns |
| 18 | 0.3414 | ns | 0.0065 | ** | 0.0001 | *** |
| 19 | 0.6171 | ns | 0.0197 | * | 0.0023 | ** |
| 20 | 0.7852 | ns | 0.0066 | ** | 0.0017 | ** |
| 21 | 0.6962 | ns | < 0.0001 | **** | < 0.0001 | **** |
| 22 | 0.0276 | * | 0.0304 | * | < 0.0001 | **** |
| 23 | 0.8672 | ns | 0.0249 | * | 0.0105 | * |
| 24 | 0.6323 | ns | 0.0576 | ns | 0.0084 | ** |
| 25 | 0.8887 | ns | 0.0217 | * | 0.0973 | ns |
| 26 | 0.2506 | ns | 0.0383 | * | 0.6914 | ns |
| 27 | 0.1940 | ns | 0.0119 | * | 0.5252 | ns |
| 28 | 0.0895 | ns | 0.0360 | * | 0.9332 | ns |
| 29 | 0.0303 | * | 0.0146 | * | 0.9669 | ns |
| 30 | 0.0552 | ns | 0.0433 | * | 0.9954 | ns |
