# Supplementary figures and images for: Electrical Stimulation of the M1 Activates Somatostatin Interneurons in the S1: Potential Mechanisms Underlying Pain Suppression
Source: eNeuro. 2025 Apr 25;12(4):ENEURO.0541-24.2025. doi: 10.1523/ENEURO.0541-24.2025 (PMC12043047; doi:10.1523/ENEURO.0541-24.2025)

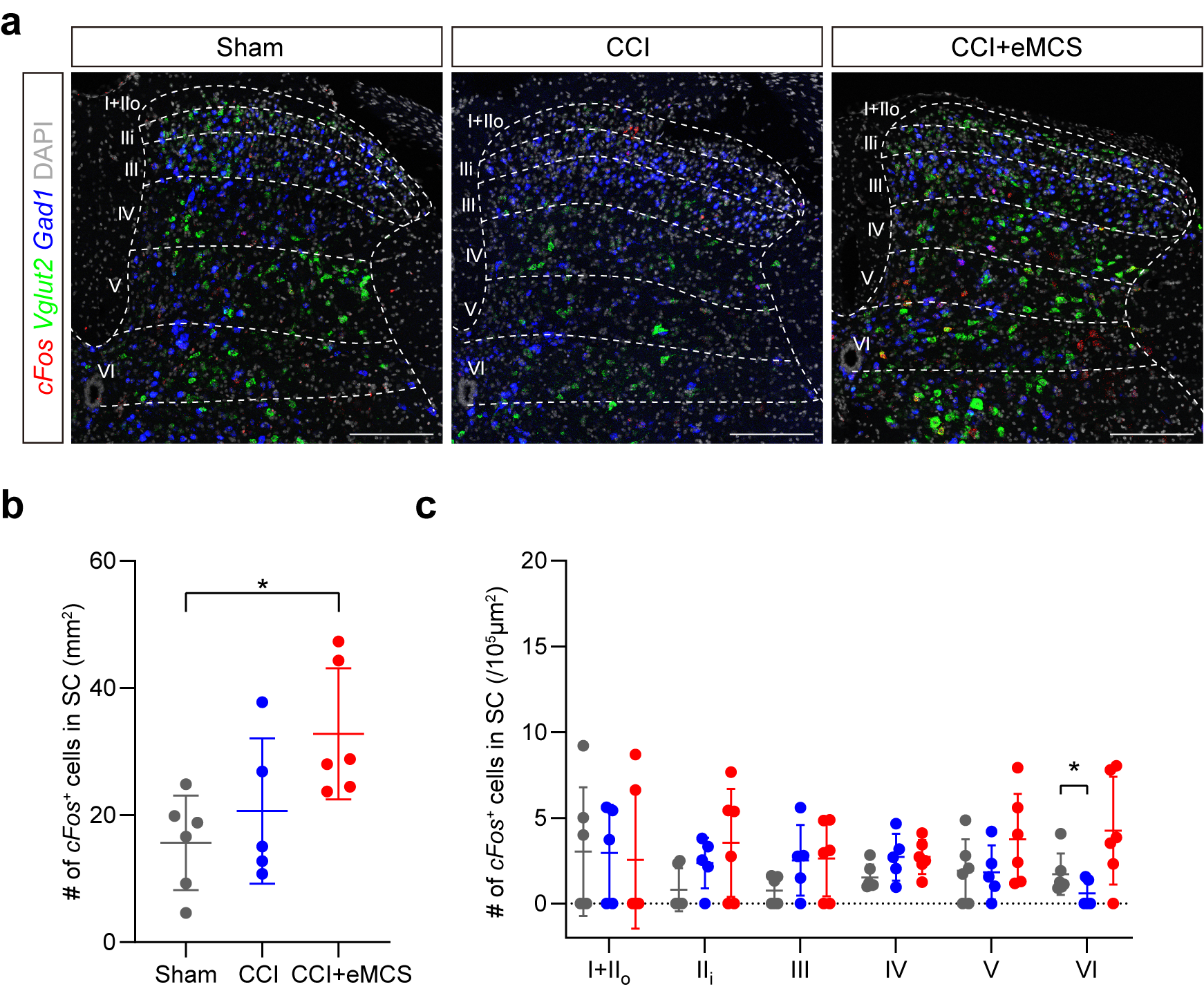

Supplement: Figure 2-1 — c-Fos expression in the lumbar region of the spinal cord. (a) Representative images of multiplex RNAscope in situ hybridization in the caudal lumbar region of the spinal cord (from left to right: sham, CCI, and CCI + eMCS). Red: c-Fos; green: Vglut2; blue: Gad1; grey: DAPI. Rexed laminae are outlined with white dashed lines. Sham: skin incision only; CCI: CCI surgery; CCI + eMCS: eMCS with CCI surgery. Scale bars: 200 µm. (b, c) Quantification of the number of c-Fos+ cells in the spinal cord. Total number of c-Fos+ cells (b) and the number of c-Fos+ cells in each Rexed lamina (c). Sham group (n = 2, 6 sections); CCI group (n = 2, 5 sections); CCI + eMCS group (n = 2, 6 sections). Lines indicate the mean ± SD. Only significant differences are indicated: *p < 0.05; one-way ANOVA followed by Tukey's test. Download Figure 2-1, TIF file. [file eneuro-12-ENEURO.0541-24.2025-s003.tif]

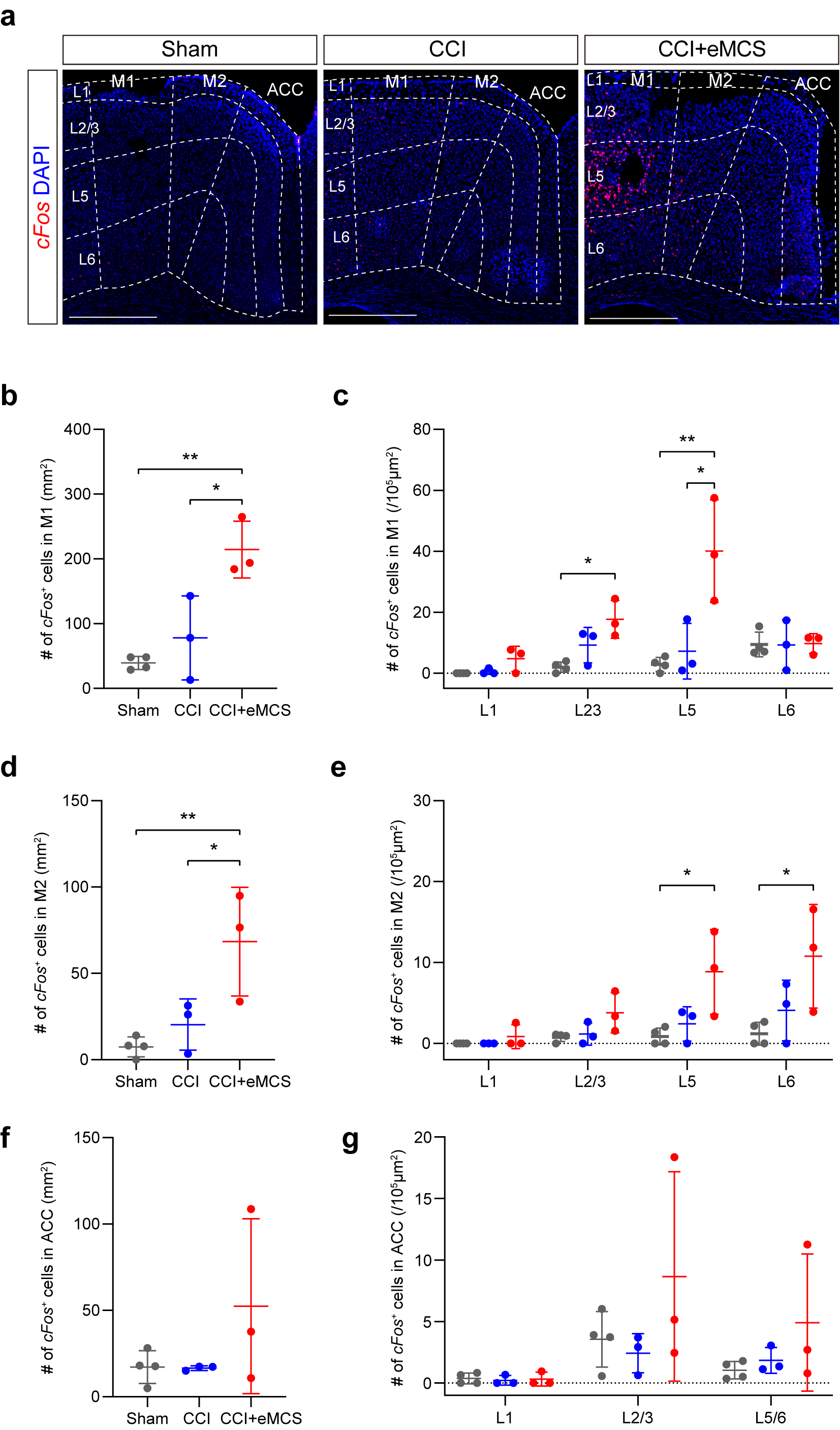

Supplement: Figure 2-2 — c-Fos expression in brain regions adjacent to the M1 region. (a) Representative images of c-Fos expression in brain regions medial to the M1 region (M2 and ACC) across groups (from left to right: Sham, CCI, and CCI + eMCS). Red: c-Fos; blue: DAPI. Cortical layers are demarcated with white dashed lines. Sham: skin incision only; CCI: CCI surgery; CCI + eMCS: eMCS with CCI surgery. Scale bars: 500 µm. (b–g) Quantification of the number of c-Fos+ cells. Sham group (n = 4); CCI group (n = 3); CCI + eMCS group (n = 3). (b, d, and f) Total number of c-Fos+ cells in the M1 (b), M2 (d), and ACC (f). (c, e, and g) Number of c-Fos+ cells in each cortical layer in the M1 (c), M2 (e), and ACC (g). Lines indicate the mean ± SD. Only significant differences are indicated: *p < 0.05, **p < 0.01; one-way ANOVA followed by Tukey's test. Download Figure 2-2, TIF file. [file eneuro-12-ENEURO.0541-24.2025-s004.tif]

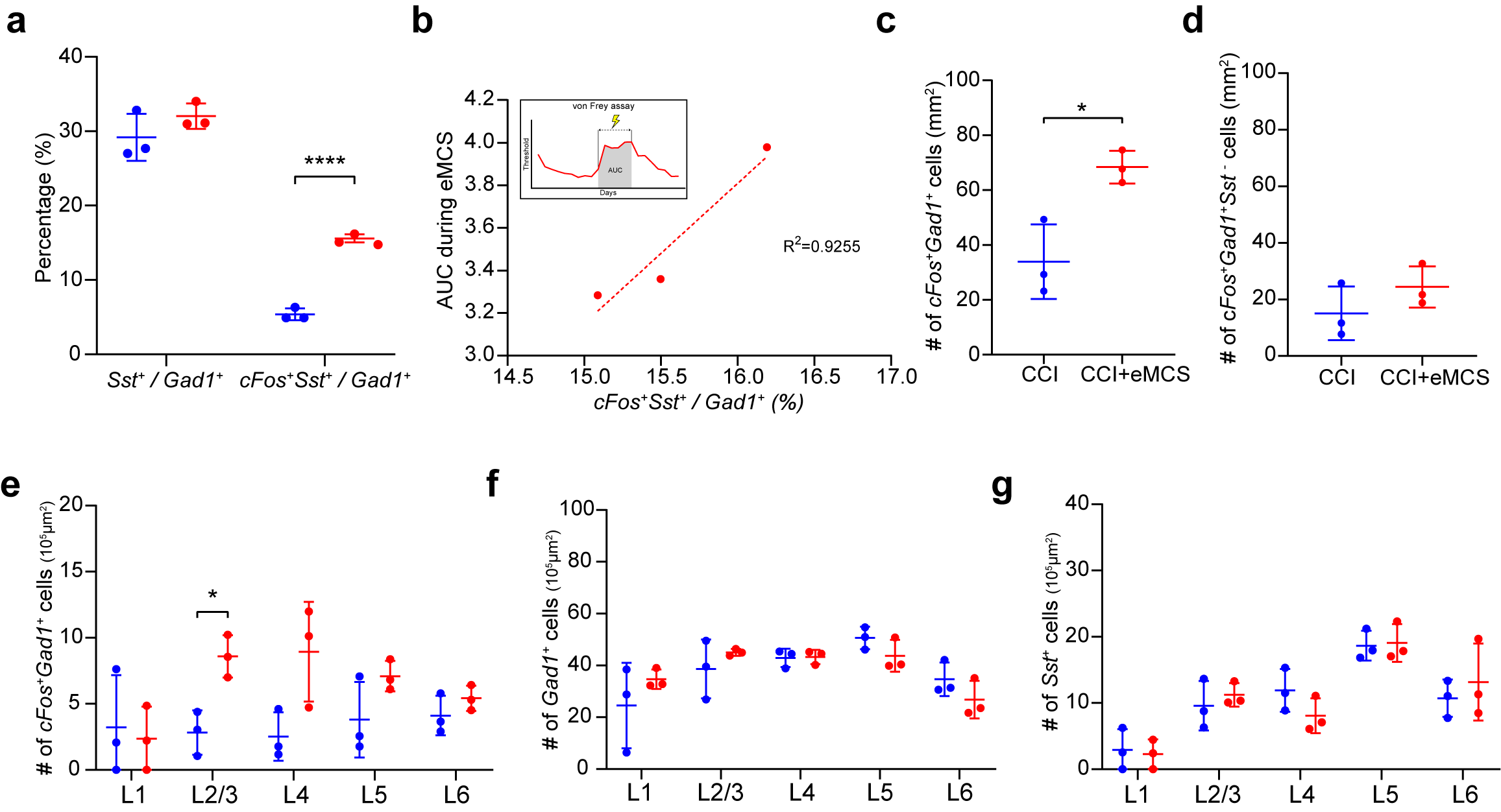

Supplement: Figure 4-1 — Layer-specific changes in neuronal activity induced by eMCS in the S1HL. (a) Quantification of Sst+ cells in the S1HL. CCI group (n=3); CCI + eMCS group (n=3). Lines indicate the mean ± SD. Only statistically significant differences are indicated: ****p < 0.0001; unpaired t-test. (b) Simple linear regression analysis between the percentage (%) of c-Fos+Sst+ cells / Gad1+ cells and the pain response (AUC of von Frey assay during eMCS). (c, d) Quantification of Gad1+ cells in the S1HL. The number of c-Fos+Gad1+ cells (c) and c-Fos+Gad1+Sst- cells (d) in the S1HL. Lines indicate the mean ± SD. Only statistically significant differences are indicated: *p < 0.05; unpaired t-test. (e–g) Distribution of c-Fos+Gad1+ cells (e), Gad1+ cells (f), and Sst+ cells (g) across the layers of the S1HL. Lines indicate the mean ± SD. Only statistically significant differences are indicated: *p < 0.05; unpaired t-test. Download Figure 4-1, TIF file. [file eneuro-12-ENEURO.0541-24.2025-s005.tif]
